# Etiology of acute undifferentiated fever in patients presenting to the emergency department of a tertiary care center in Karachi, Pakistan

**DOI:** 10.12669/pjms.36.6.2334

**Published:** 2020

**Authors:** Noman Ali, Nadeem Ullah Khan, Shahid Waheed, Syed Mustahsan

**Affiliations:** 1 Noman Ali, Senior Instructor, Department of Emergency Medicine, Aga Khan University Hospital, Karachi, Pakistan; 2 Nadeem Ullah Khan Associate Professor, Department of Emergency Medicine, Aga Khan University Hospital, Karachi, Pakistan; 3 Shahid Waheed Senior Instructor, Department of Emergency Medicine, Aga Khan University Hospital, Karachi, Pakistan; 4 Syed Mustahsan Resident, Department of Emergency Medicine, Aga Khan University Hospital, Karachi, Pakistan

**Keywords:** Undifferentiated fever, Emergency department, Malaria, Dengue

## Abstract

**Objective:**

Our study aimed at identifying the characteristics and etiology of various causes of acute undifferentiated fever in patients presenting to the emergency department of a tertiary care hospital.

**Methods:**

This was a retrospective study conducted at the department of emergency medicine, Aga Khan University Hospital from January to June 2016. Adult patients presenting to Emergency department with acute undifferentiated fever were enrolled. Descriptive statistics were calculated in terms of mean±SD for continuous variables like age of the patients and duration of fever, whereas frequency and percentage were computed for categorical variables like gender and causes of fever.

**Results:**

A total of one hundred and fifty five patients were included. Out of these 97 (62.6%) were males and 58 (37.4%) were females. Most patients (25.2%, n= 39) were diagnosed as malaria followed closely by dengue fever (n=33, 21.3%) and then enteric fever (n= 10, 6.5%). while 41.9% (n=65) were diagnosed as suspected viral fever based on clinical judgment and inconclusive laboratory results.

**Conclusion:**

Malaria was found to be the most common confirmed cause of acute undifferentiated fever followed by dengue and enteric fever. The provision of accurate epidemiological data will enable resources to be directed towards key areas and will be of practical importance to clinicians.

## INTRODUCTION

Infectious diseases are among the leading causes of morbidity and death in developing countries like Pakistan. According to World Health Organization data Pakistan is facing challenges regarding the health system. The country has the 5th highest tuberculosis burden in the world, and focal geographical area of malaria endemicity. Around 25-30% of the Emergency Departments revisits are related to infections.^1^ Fever is one of the most common presenting complaints at emergency departments (EDs). It can attribute to a wide range of clinical disease from self-limiting viral illness to life threatening conditions like sepsis which results in mortality and morbidity leading admission to intensive care unit or prolonged hospital stay.

Acute undifferentiated fever is a common cause of visits to health care providers in South Asian countries in particular. It is defined as fever without a focus of infection on initial history and physical examination. It represents a major burden of disease with diagnostic and therapeutic challenges. Malaria, dengue and typhoid are common infectious causes of acute undifferentiated fever, causing considerable economic burden and prescription of empirical broad spectrum antibiotics with little evidence.^2^

There are various possible causes of acute undifferentiated fever including infections, connective tissue disorder malignancies and number of miscellaneous conditions that sometimes remain undiagnosed despite of several laboratory investigations. A key clinical question is deciding whether infection is likely enough to warrant antimicrobial administration. A detailed history and physical exam, the past medical history, current medications (e.g. chemotherapy, glucocorticoids), and recent use of antibiotics may help shape the pre-test probability of an infectious source of fever. However, it is common to need adjunctive laboratory testing or radiographic imaging to further evaluate the source of the fever. For an adequate risk stratification of febrile patients, thorough information about local epidemiology is required, and risk factors associated with adverse clinical outcomes have to be identified.

Although the underlying conditions that are the root causes of fever vary considerably, this requires a systematic approach regardless of the underlying condition. In most parts of the world fever is considered as a strong rationale for the institution of antibiotics in ED settings which has led to increased antibiotic resistance over the years. Self-medication has become very common among febrile subjects in many developing countries. Undifferentiated fever remains undiagnosed in many cases despite extensive investigations. Some of them resolve spontaneously while some remain undiagnosed.^3^

In Pakistan, fever is one of the most common reasons for seeking medical attention, but there is limited information on the incidence of specific infections presenting to ED settings. The burden of some infections like malaria, dengue, enteric fever and pneumonia seems to be more as compared to other infections. The provision of accurate epidemiologic data will enable resources to be directed towards key areas and will be of practical importance to clinicians. Therefore, the aim of this study was to describe the characteristics and etiology of undifferentiated fever among adult patients in a tertiary care hospital.

## METHODS

This was retrospective study conducted at Aga Khan University Hospital (AKUH) over the period of six months from January 1 to June 30, 2016.

### Inclusion criteria

The study include patients of age ≥16 years, presenting to the emergency department with axillary temperature of ≥ 38°C with duration of fever ≤ 15 days without any evident focus of infection following initial clinical evaluation.

### Exclusion criteria

Patients who were presented with any signs of localized infection or on antibiotics for more than 24 hours were excluded. Patients who had malignancies or are on immunosuppressant were also excluded.

Demographic and clinical data including signs and symptoms were recorded from medical record files of the patients using a standardized form. The evaluation of the patient, diagnostic tests and treatment was entirely at the discretion of treating physician.

### Data Analysis

Descriptive statistics were calculated in terms of mean±SD of age of the patients and duration of fever respectively, whereas frequency and percentage was computed for gender and causes of fever like malaria, typhoid, urinary tract infections, dengue fever etc. Stratification was done with regards to age, gender and duration of fever to see the effect of these on outcome variables. P value considered significant at <0.05.

### Diagnostic Criteria

### Malaria

Positive malaria parasite on blood smear or positive malaria parasite immunochromatography.

### Urinary tract infection

Presence of bacterial colony count of greater than or equal to1000 colony-forming units per ml on urine culture.

### Dengue fever

Positive dengue antigen or IgM antibody.

### Enteric fever

Blood culture positive for Salmonella typhi or Salmonella paratyphi.

### Pneumonia

Finding of air-space shadowing on chest radiograph

Ethical approval for the study was obtained from the ethical review committee (3740-EM-ERC-15, dated October 20, 2015) of the Aga Khan University Hospital, Pakistan (AKU).

## RESULTS

A total of 155 patients were enrolled in the study during the period of January 1 to June 30, 2016. Of these, 97 (62.6%) were males and 58 (37.4%) were females. The mean age was 35.57±14.17. Triaging of the patients was done using a five level ED triage algorithm known as Emergency Severity Index (ESI). Most of the patients fall in the category those who required urgent care (n= 140, 90.3%) followed by (12, 7.7%) in P2 (critical), only three patients in P4 (walk-in) category (1.9%). None of the patient presented in either P1 or P5 category. Duration of fever ranged from 1-15 days and most of the patients presented with fever less than or equal to five days (n= 103, 66.6%) and rest of the patients presented with fever between 6-15 days as shown in Table-I.

**Table-I:** Basic demographic characteristics of patients.

	N	%
***Sex***		
Male	97	62.6
Female	58	37.4
Age in years (Mean)	35.57 ±14.17	
15-20	29	18.7
21-30	39	25.2
31-40	34	21.9
41-50	26	16.8
≥51	27	17.4
***Duration of fever (days)***		
≤ 5	103	66.6
6-10	47	22.5
11-15	5	3.2
***Triage Category***		
P2 (Critical)	12	7.7
P3 (Urgent)	140	90.3
P4 (Walk-in)	3	1.9

The initial laboratory works up including complete blood count (CBC), malaria parasites (MP) and malarial parasite immunochromatography (MPICT) were performed in all patients. Blood culture and dengue testing (antigen and antibody) were performed in selected patient as per clinician judgment. We were able to confirm cause of undifferentiated fever in more than half of patient (n= 90, 58%) of patient, while (n=65, 42%) had inconclusive laboratory results and were diagnosed as suspected viral fever based on clinical judgment

**Fig.1:**
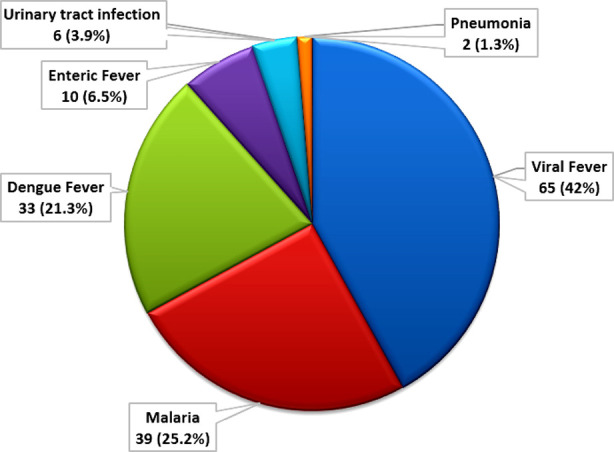
Causes of fever.

All patients with suspected viral fever presented with fever of varying duration. Most of the patients had associated symptoms like nasal congestion and dry cough. CBC, MP and MPICT were performed in all cases to rule out malaria as a possible cause. All the patients were discharged on symptomatic treatment like antipyretics and analgesics (e.g. acetaminophen). Of those patient in which confirmed diagnosis were made, most patients (n= 39, 25.2%) were diagnosed as malaria followed closely by dengue fever (n=33, 21.3%) and then enteric fever (n= 10, 6.5%) as shown in Fig.1.

Malaria was found to be the most common confirmed cause of acute undifferentiated fever. Out of 39 patients with malaria, peripheral films of (n= 32, 82%) patients showed plasmodium vivax and MPICT showed mixed positive results while in (n= 7, 18%), peripheral film showed plasmodium falciparum.

Dengue fever was found to be the second most common confirmed cause of acute undifferentiated fever. Out of 33 patients with dengue the antigen was positive in (n= 10, 30%) patients and dengue IgM was positive in (n=23, 70%) patients.

Enteric fever was the third leading confirmed cause of acute undifferentiated fever in our study (n=10, 7%).Blood culture of eight out of 10 patients showed salmonella typhi, while two patients had salmonella paratyphi. All of them were sensitive to third generation cephalosporin.Moreover, out of 155 patients only eight were found to have systemic focus of infection on laboratory test and radiological imaging without clinical finding/correlation. Of these eight patients, six (3.9%) had urinary tract infection and two (1.3%) had pneumonia.

Causes of fever were compared with age categories, gender and duration. It was found that causes of fever were statistically different for males and females (p value 0.004) and duration of fever presentation (< 7 days versus 7-15 days, p value 0.013) as depicted in Table-II.

**Table-II:** Cause of fever categorized by age, gender and duration of fever.

Percentage of disease by age			

	16-34 years	≥35 years	P-value
Dengue	21 (25.9%)	12 (16.2%)	0.204
Malaria	20 (24.7%)	19 (25.7%)	
Pneumonia	0	2 (2.7%)	
Enteric Fever	5 (6.2%)	5 (6.8%)	
Urinary Tract Infection (UTI)	5 (6.2%)	1 (1.4%)	
Viral Fever	30 (37%)	35 (47.3%)

Percentage of disease by gender			

	Female	Male	P-value

Dengue	16 (27.6%)	17 (17.5%)	0.004
Malaria	9 (15.5%)	30 (30.9%)	
Pneumonia	0	2 (2.1%)	
Enteric Fever	7 (12.1%)	3 (3.1%)	
Urinary Tract Infection (UTI)	5 (8.6%)	1 (1.0%)	
Viral Fever	21 (36.2%)	44 (45.4%)

Duration of Fever			

	<7 days	7-15 days	P-value

Dengue	24 (20.8%)	9 (22.5%)	0.013
Malaria	27 (23.5%)	12 (30.0%)	
Pneumonia	2 (1.7%)	0	
Enteric Fever	3 (2.6%)	7 (17.5%)	
Urinary Tract Infection (UTI)	5 (4.4%)	1 (2.5%)	
Viral Fever	54 (47%)	11 (27.5%)	

## DISCUSSION

Acute undifferentiated fever (AUF) is the clinical illness in which, it is difficult to find out causative agents and once detected can be treated on the line of etiological agent. The predominance of infections caused by mosquito’s bite is usually observed during post-monsoon period. Several studies have shown increased in number of cases from September to November.^4^ Post-monsoon environment provides favorable breeding places for the mosquitoes. Attributing a detected pathogen to clinical diagnosis or to the cause of AUF is a major issue; however, majority of the AUF can be reliably predicted using proper history, good physical examination and laboratory tests.^5^ Proper protocol use for AUF can also help in the appropriate use of antibiotics as well as investigations. This reduces cost and resistance to antibiotics.

Our study highlighted various etiologies and clinical presentations of acute undifferentiated fever in an ED of a tertiary care center of low and middle income country. The study confirmed etiology in almost 58% of cases. Furthermore, this study also reveals heavy burden of tropical infections like malaria, dengue and typhoid in our setting. A similar study was conducted by Singh R et al. from the region of Uttarakhand, India also showed that dengue; malaria and enteric fever are the common etiological agents of acute febrile illness.^6^

Most of the patients (41.9%) were categorized as suspected viral fever followed by malaria (25.2%) and dengue fever (21.3%). Our results are similar to those found in other tropical regions of the developing world, although the relative frequency of specific pathogens varies from place to place. Male predominance was noticed in malaria and dengue fever. This may be due to male gets more exposed to the outer environment.

It was estimated that in 2018 around 228 million cases of malaria occurred worldwide, compared with 251 million cases in 2010 and 231 million cases in 2017. Most malaria cases in 2018 were in the World Health Organization (WHO) African Region (213 million or 93%), followed by the WHO South-East Asia Region with 3.4% of the cases and the WHO Eastern Mediterranean Region with 2.1%. Nineteen countries in sub-Saharan Africa and India carried almost 85% of the global malaria burden. Plasmodium falciparum is the most prevalent malaria parasite in the WHO African Region, accounting for 99.7% of estimated malaria cases in 2018, as well as in the WHO South-East Asia Region (50%), the WHO Eastern Mediterranean Region (71%) and the WHO Western Pacific Region (65%). Globally, 53% of the *P. vivax* burden is in the WHO South-East Asia Region, with the majority being in India (47%). *P. vivax* is the predominant parasite in the WHO Region of the Americas, representing 75% of malaria cases.^7^

Malaria occurs throughout most of the tropical regions of the world, with plasmodium *falciparum* causing the major burden of disease, followed by *Plasmodium Vivax.^8^ Plasmodium malariae* is uncommon and is found in most endemic areas, especially in sub-Saharan Africa. *Plasmodium ovale*, less common, is relatively rare outside of Africa and comprises <1 percent of isolates. *Plasmodium knowlesi*, similar morphologically to *Plasmodium malariae*, has been recognized by molecular methods in patients in Malaysia, the Philippines, Thailand, and Myanmar.^9^

In Pakistan malaria continues to be a serious public health problem. Around 2.6 million malaria cases were reported nationwide in 2008, with a mortality rate of 50,000 per year.^10^ *Plasmodium Vivax* and *P. falciparum* are the two major *Plasmodium* species found in Pakistan, with *P. Vivax* predominating in most regions.^11^,^12^ Our study also shows similar results as malaria was found to be the second most common cause of acute undifferentiated fever and most of these were due to *Plasmodium Vivax*.

The most recent outbreak on dengue fever was reported in 2019 by the Ministry of National Health Services, Regulations and Coordination (MNHSR&C) of Pakistan. Total of 998 new cases of dengue fever during epidemiologic week 48 (25 November – 1 December) of 2019. Out of these new cases, 789 (79%) were reported from Sindh and 209 (20%) from the rest of the regions in Punjab, Islamabad, Khyber Pakhtunkhwa (KP), Baluchistan, Azad Jammu and Kashmir (AJK) and tribal districts of KP. Within Sindh, 14 443 (93%) of the total 15 521 cases are reported from Karachi alone.^13^ In our study, dengue is the third leading cause of acute undifferentiated fever (21.3%). We usually perform dengue antigen test if a patient present within five days of symptom onset like high grade fever associated with myalgias and arthralgias, after five days we perform dengue IgM. The reason we were able to detect the malaria and dengue in most of the cases may be that the diagnostic test is available and done most of the times when patient presents with similar symptoms. Enteric fever (typhoid) continues to be endemic in poor countries, such as Pakistan, where it represents one of the leading causes of morbidity and mortality in the country.^14^ Enteric fever is caused by *Salmonella. Typhi*. The bacterium is transmitted by fecal-oral route, through contaminated water or food source. Nowadays extensive drug resistant typhoid is emerging as a global challenge for our country and our general physicians. In our study we identified enteric fever (typhoid) as fourth common cause of AUF.

### Limitations of the study

This study has several limitations. Firstly, we only reported the current clinical practice of diagnosis of acute undifferentiated fever and did not make an exhaustive search into all the causes of fever since viral studies are not available in our institute and it would have increased the cost to the patient. Secondly, the study included only adult patients while children would expect to have different fever etiologies due to exposure and immunity. Hence, the pattern of fever etiology found in this study would not be representative for the causes of fever in general population. Thirdly in this study we were unable to follow the patient diagnosed with suspected viral fever to find out if these patients did get better with symptomatic treatment or later were found to have some other diagnosis. In our country lack of primary care physician led to lost to follow up of these patients. Lastly the study duration was six months and seasonal variation of infectious causes could have affected our results. Further studies are needed, both community and hospital based including the adult as well as pediatric population in order to provide more evidence based information about the prevalence of different etiologies of undifferentiated fever in tropical countries like Pakistan.

## CONCLUSION

Despite of all limitations, our study clearly revealed that predominant cause of acute undifferentiated fever in our region was malaria, followed by dengue and typhoid. Our study highlighted that finding a cause of acute undifferentiated fever in 42% of cases is challenging in emergency setting. Of the confirmed diagnosis the most common was Malaria followed by dengue and Enteric fever. Forty-two percent of cases were presumed to have viral infection based on clinical judgment and inconclusive laboratory test.

### Availability of data and materials

The datasets used and/or analyzed during the current study are available from the corresponding author on reasonable request.

### Authors’ Contributions:

**NA and NUK** conceived the idea and developed the initial draft of the paper.

**SW and SM** collected the data and did the data entry.

**NUK** analyzed and interpreted the data.

**NA** Prepared the final draft and is accountable for the accuracy and integrity of work.

All authors read and approved the final manuscript.
